# Identification and Evaluation of Antioxidant and Anti-Aging Peptide Fractions from Enzymatically Hydrolyzed Proteins of *Spirulina platensis* and *Chlorella vulgaris*

**DOI:** 10.3390/md23040162

**Published:** 2025-04-08

**Authors:** Baran Masoumifeshani, Abdolmohammad Abedian Kenari, Ignacio Sottorff, Max Crüsemann, Jamshid Amiri Moghaddam

**Affiliations:** 1 Aquaculture Department, Natural Resources and Marine Science Faculty, Tarbiat Modares University, 46417-76489 Noor, Mazandaran, Iran; h.masoomi@modares.ac.ir; 2 Institute for Pharmaceutical Biology, University of Bonn, 53115 Bonn, Germany; isot@uni-bonn.de (I.S.); cruesemann@em.uni-frankfurt.de (M.C.); 3 Institute of Pharmaceutical Biology, Goethe University Frankfurt, 60438 Frankfurt am Main, Germany; 4 Institute of Biotechnology, RWTH Aachen University, 52074 Aachen, Germany

**Keywords:** antioxidant activity, bioactive peptides, anti-aging function, enzymatic hydrolysis, *Spirulina platensis*, *Chlorella vulgaris*

## Abstract

Microalgae are a promising source of bioactive compounds, particularly proteins and peptides, with potential applications in skin health and the cosmetic industry. This study investigated the antioxidant and anti-aging properties of peptide fractions derived from *Spirulina platensis* and *Chlorella vulgaris*. Both microalgae were cultivated, and their proteins were subsequently extracted, enzymatically hydrolyzed with alcalase, and fractionated through ultrafiltration. Alkaline extraction yielded 82% protein from *S. platensis* and 72% from *C. vulgaris*. Enzymatic hydrolysis predominantly yielded <3 kDa peptides, which exhibited strong antioxidant activity reaching 78% for 2,2-diphenyl-1-picrylhidrazol (DPPH), 82% for 2,2′-azinobis-3-etilbenzothiazoline-6-sulfonic acid (ABTS), and 74% for ferric reducing antioxidant power (FRAP), with IC_50_ values as low as 23.44 µg/mL for ABTS inhibition in *C. vulgaris*. These peptides also significantly inhibited skin-aging enzymes, showing 84% inhibition of elastase, 90% of collagenase, and 66% of tyrosinase. Mass spectrometry and GNPS molecular networking of the <3 kDa fraction identified several di- and tri-peptides, including Lys-Val, Val-Arg, His-Ile, Lys-Leu, Ile-Leu, and Leu-Phe, Tyr-Phe, and Leu-Gly-Leu, potentially contributing to these bioactivities. These findings suggest that the enzymatic hydrolysis of *S. platensis* and *C. vulgaris* proteins provides a sustainable and natural source of bioactive peptides for antioxidant and anti-aging applications in food, pharmaceutical, and cosmetic industries.

## 1. Introduction

Microalgae represent a promising and sustainable source of bioactive compounds, particularly proteins and amino acids, with significant implications for skin health and applications in the cosmetic, hygienic, and pharmaceutical industries [1,2]. The growing consumer preference for natural, cost-effective, and safe dietary supplements has further fueled interest in microalgae-derived products, especially given their potential for disease prevention and treatment [3]. Microalgae’s inherent protective mechanisms against free radicals and reactive oxygen species (ROS) underscore their value in diverse industrial sectors, with the cosmetic and hygienic industry being a prime example. Specifically, the potential of microalgae to produce compounds that enhance skin health and beauty is a key driver of current research [4].

The enzymatic hydrolysis of microalgal proteins offers a powerful approach to unlocking and enhancing their bioactivity by releasing peptides with specific biological functions [5]. These bioactive peptides, typically 2–20 amino acids long, are often inactive within the parent protein but become activated upon hydrolysis, exhibiting a range of physiological effects. Marine organisms, particularly algae, are recognized as rich sources of these peptides, offering advantages over synthetic alternatives due to their high bioactivity and potentially fewer side effects [6,7]. Microalgae-derived peptides have demonstrated a diverse array of functional properties, including immune stimulation, anti-aging, antibacterial, antihypertensive, and, importantly, antioxidant activities. These activities are intricately linked to factors such as the protein source, hydrolysis conditions, degree of hydrolysis, molecular weight, amino acid composition, and the specific sequence and positioning of the peptide within the protein [8]. The growing recognition of peptides’ potential in cosmetics and skincare further emphasizes their importance [9]. Recent studies have explored the combined use of enzymatic hydrolysis and filtration to enhance the bioactivity of microalgal hydrolysates, demonstrating that this approach can increase protein content and bioactivities. For example, hydrolysates generated from mixed *Chlorella* sp. and *Scenedesmus* sp. using sequential hydrolysis with Viscozyme and alcalase, followed by membrane filtration, exhibited anti-angiotensin-I-converting enzyme (ACE-1), anti-amylase, and antioxidant activities, as well as techno-functional properties suitable for food ingredients [10]. This underscores the potential of optimized hydrolysis and filtration strategies to unlock diverse bioactivities from microalgae.

*Spirulina platensis* and *Chlorella vulgaris* are two microalgae species that have gained considerable attention due to their nutritional and therapeutic potential. *S. platensis*, a photosynthetic cyanobacterium, is valued as a supplement and food additive due to its high protein content and diverse array of beneficial compounds, including pigments, beta-carotenes, polysaccharides, and peptides. Its high protein content and favorable amino acid profile position *S. platensis* as a potential substitute for animal proteins [7]. Similarly, *C. vulgaris* is a rich source of essential nutrients, including proteins, fats, vitamins, and carbohydrates. Its rapid growth and high biomass production make it particularly attractive for cosmetic, hygienic, and pharmaceutical applications, especially due to its polysaccharide content [11]. The high protein content and established therapeutic properties of both *S. platensis* and *C. vulgaris* suggest their potential as valuable sources of bioactive peptides.

A critical aspect of cellular health, particularly in the context of aging, is the management of oxidative stress. ROS, while essential for certain cellular processes [4], can cause significant damage to cellular components when produced in excess. Antioxidants, whether endogenously produced or obtained through supplementation, play a crucial role in scavenging ROS and mitigating oxidative stress, thus contributing to skin health by potentially reducing wrinkles and inflammation [12]. The search for natural antioxidants, which are perceived as less hazardous than some synthetic compounds, has intensified [3]. The marine environment is a rich source of bioactive compounds, including antioxidant peptides. For instance, studies have identified and characterized antioxidant peptides from various marine organisms, such as miiuy croaker swim bladders [13], skipjack tuna (*Katsuwonus pelamis*) skins [14], Siberian sturgeon (*Acipenser baerii*) cartilages (specifically antioxidant collagen peptides) [15], and the protein hydrolysate of skate (*Raja porosa*) cartilage [1]. These studies highlight the diverse potential of marine resources for deriving natural antioxidants. Algae are a rich source of such antioxidants, including phycobiliproteins, phlorotannins, carotenoids, sulfated polysaccharides, siphonein, mycosporine-like amino acids, and vitamins C and A, all known for their skin-soothing and cleansing properties [16].

Aging, an inevitable biological process, presents a major global health challenge. Strategies to mitigate the effects of aging and age-related diseases, including the use of bioactive compounds, are of paramount importance [17,18]. A hallmark of skin aging is the progressive degradation of extracellular matrix (ECM) proteins, such as collagen, elastin, and laminin, which are crucial for maintaining skin structure and elasticity and influencing cell proliferation and differentiation [19]. This degradation leads to the release of matricryptins, peptides that regulate skin structure, elasticity, and cell adhesion/signaling [20]. Consequently, the cosmetic industry is increasingly focused on incorporating bioactive agents, including peptides, growth factors, and antioxidants, into anti-aging products. Peptides, in particular, are highly sought after for their potential to reduce wrinkles and combat the visible signs of aging [21].

Given the established potential of antioxidant peptides from diverse marine sources, including the aforementioned examples, this study focuses on exploring the antioxidant and anti-aging potential of peptide fractions derived from enzymatically hydrolyzed proteins of the seaweeds *S. platensis* and *C. vulgaris*. This study addresses a critical gap in current research by conducting a comprehensive investigation into the production and characterization of bioactive peptides from the enzymatically hydrolyzed proteins of *S. platensis* and *C. vulgaris*. Specifically, we aim to fractionate the hydrolyzed protein mixtures and evaluate the antioxidant and inhibitory effects on key enzymes involved in skin aging of the resulting peptide fractions. Furthermore, we combined high-performance liquid chromatography–tandem mass spectrometry (HPLC-MS/MS) with molecular networking to identify and characterize the peptide components within the active fractions. By systematically identifying the most potent fractions, this research will pave the way for the development of novel anti-wrinkle and anti-aging skincare products with demonstrated efficacy.

## 2. Results and Discussion

### 2.1. Microalgae Growth

The growth patterns of *S. platensis* and *C. vulgaris* were assessed by monitoring optical density, cell counts, and dry weight over the cultivation period in liquid media. The growth rates of the *S. platensis* and *C. vulgaris* are shown in Figure S1A–D. *S. platensis* was harvested on day 12 of cultivation when the optical density and the cell count reached 1 and 1.34 × 10^7^ cells/mL, respectively. The harvested *S. platensis* biomass had a dry weight of 1 g/L, indicating that the dry weight constituted 0.10% of the wet weight. For *C. vulgaris*, the cells were collected on day 14 of cultivation when the optical density reached 1 and the cell count reached 1.29 × 10^7^ cells/mL, respectively. The dry weight yield for this microalga was 0.80 g/L, representing 0.08% of the wet biomass, as shown in Table 1.

The chemical composition of the microalgae dry materials, including protein, fat, moisture, ash, and carbohydrates, was measured (see Table 1). *S. platensis* showed 64 ± 1.45% protein, 10 ± 0.88% fat, 9 ± 0.72% moisture, 11 ± 0.50% ash, and 6% carbohydrates. For *C. vulgaris*, the composition was 54 ± 0.88% protein, 9 ± 0.90% fat, 5 ± 0.60% moisture, 14 ± 0.78% ash, and 18% carbohydrates. Additionally, the fat content after defatting of the microalgae powder was observed to be 2.36 ± 0.23% in *S. platensis* and 3.26 ± 0.35% in *C. vulgaris*. Statistical analysis of the raw powder and protein isolate of the microalgae showed that the protein content in both cases was significantly higher (*p* < 0.05) in *S. platensis* than in *C. vulgaris* (Table 1). These protein contents align with previously reported ranges for these microalgae [22,23,24], confirming their status as protein-rich biomass suitable for peptide production.

### 2.2. Protein Isolation, Enzymatic Hydrolysis, and Peptide Purification

The protein content for the alkaline isolate of *S. platensis* was 82 ± 0.92% and 72 ± 0.65% for *C. vulgaris*. This represents a significant increase (*p* < 0.05) in protein content after isolation compared to the raw material for both microalgae (Table 1). After enzymatic protein hydrolysis with alcalase, the degree of hydrolysis for *S. platensis* was about 33 ± 0.70%, with a peptide chain length of 3.07 ± 0.08 amino acid residues. Similarly, the degree of hydrolysis for *C. vulgaris* was 30 ± 1.19%, with a peptide chain length of 3.33 ± 0.02 amino acid residues. There was no statistically significant difference in the degree of hydrolysis between the two species (*p* ≥ 0.05); however, the peptide chain length in *C. vulgaris* was significantly higher (*p* < 0.05) than in *S. platensis* (Table 1). These values, while indicative of substantial peptide production, differ from some previous reports [25,26], likely due to variations in hydrolysis conditions, the enzyme type, and the substrate [27,28,29]. The use of alcalase, known for its high hydrolysis efficiency, facilitated the production of a high yield of peptides. Additionally, the observed differences in peptide chain length between the two microalgae may be attributed to differences in their protein structures and amino acid compositions.

After enzymatic hydrolysis, peptides were purified using 10 and 3 kDa filters. The amount of peptides in the fraction smaller than 3 kDa was significantly higher (*p* < 0.05) than in the fraction larger than 10 kDa and the fraction between 3 and 10 kDa. For *S. platensis*, 1 g of the hydrolyzed protein yielded 100 mg of peptides larger than 10 kDa, 200 mg between 3 and 10 kDa, and 650 mg smaller than 3 kDa. For *C. vulgaris*, these amounts were 100, 150, and 700 mg, respectively (Table 1). The predominance of <3 kDa peptides in both species, as revealed using ultrafiltration, shows a successful hydrolysis of larger proteins into smaller, potentially bioactive peptides. This finding is consistent with previous studies [9,30,31] and allowed for the precise determination of bioactivity in different molecular weight fractions. However, a study using a dual-enzyme system (Viscozyme and alcalase) on mixed *Chlorella* sp. and *Scenedesmus* sp. cultures revealed a higher proportion of peptides < 3 kDa post-filtration [10], suggesting that enzymatic strategies significantly impact peptide size distribution.

### 2.3. Antioxidant Activity of Peptide Fractions

The antioxidant potential of the peptide fractions was evaluated using DPPH, ABTS, and FRAP assays. The antioxidant activity of three fractions, including F1: fraction smaller than 3 kDa, F2: fraction between 3 kDa and 10 kDa, and F3: fraction larger than 10 kDa, were tested at concentrations of 5, 10, 20, 40, and 80 µg/mL. Both *S. platensis* and *C. vulgaris* fractions exhibited significant antioxidant activities. Both peptide concentration and molecular weight significantly influenced the antioxidant activity of *S. platensis* and *C. vulgaris* fractions, as demonstrated by the DPPH assay (*p* < 0.05). Tables S1 and S2 show the antioxidant activity of peptide fractions extracted from *S. platensis* and *C. vulgaris*, including IC_50_ values for each fraction and assay.

**Figure 1:**
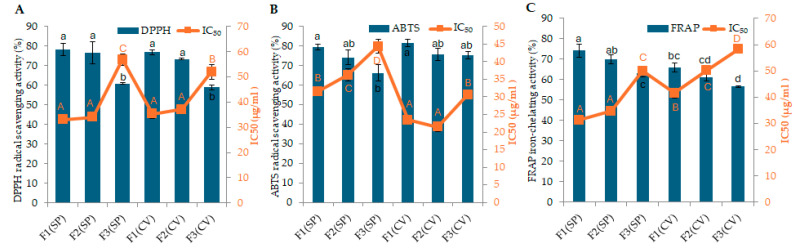
Assessment of antioxidant activities and IC_50_ values of peptide fractions (80 µg/mL) of *S. platensis* (SP) and *C. vulgaris* (CV). Fractions are defined as follows: F1 (<3 kDa), F2 (3–10 kDa), and F3 (>10 kDa): (**A**) DPPH antioxidant activity, (**B**) ABTS antioxidant activity, and (**C**) FRAP iron-chelating activity. Data are presented as the mean ± standard deviation of triplicate measurements. Different letters above bars or squares indicate statistically significant differences between mean values of fractions within each assay (*p* < 0.05). For comparison, the positive standard ascorbic acid (80 µg/mL) exhibited the following: DPPH radical scavenging activity of 91 ± 2.29% with an IC_50_ of 20.13 ± 1.31 µg/mL; ABTS radical scavenging activity of 91 ± 2.29% with an IC_50_ of 24.92 ± 0.54 µg/mL; and FRAP iron-chelating activity of 87 ± 0.24% with an IC_50_ of 7.98 ± 0.69 µg/mL.

In *S. platensis*, the highest antioxidant activity inhibition was observed in fractions 1 and 2 at a concentration of 80 µg/mL, as well as in fraction 1 at a concentration of 40 µg/mL. These values were 78% ± 3.15, 77% ± 5.65, and 73 ± 2.89%, respectively (*p* ≥ 0.05). The IC_50_ values for *S. platensis* fractions in the DPPH assay were 33.11 µg/mL (F1), 33.99 µg/mL (F2), and 56.87 µg/mL (F3), indicating that smaller molecular weight peptide fractions (F1 and F2) exhibited greater antioxidant potency (Figure 1A). Additionally, the highest antioxidant activity inhibition in *C. vulgaris* was observed in fractions 1 and 2 at a concentration of 80 µg/mL. These values were 77 ± 1.32% and 73 ± 0.37%, respectively (*p* < 0.05). The IC50 values for *C. vulgaris* fractions in the DPPH assay were 35.36 µg/mL (F1), 37.21 µg/mL (F2), and 52.04 µg/mL (F3), further supporting the trend of higher activity in smaller peptide fractions. The standard sample, ascorbic acid, showed an antioxidant activity of 91 ± 2.29% at a concentration of 80 µg/mL, with an IC_50_ value of 20.13 µg/mL. A comparison between the two microalgae species showed that there was no statistically significant difference between fractions one and two (*p* < 0.05), nor between fraction three in both species (*p* < 0.05). However, overall, fractions 1 and 2 (smaller than 10 kDa) exhibited significantly greater activity (*p* < 0.05) compared to the fraction 3 (larger than 10 kDa) in both species, as shown in Figure 1A.

The values of the ABTS assay are presented in Tables S1 and S2. The values revealed that the ABTS radical scavenging activity of both algae is concentration- and size-dependent (*p* < 0.05). The highest inhibition activity for *S. platensis* was observed in fractions 1 and 2 at a concentration of 80 µg/mL, with values of 80 ± 1.51% and 74 ± 3.70%, respectively (*p* ≥ 0.05). The IC_50_ values for *S. platensis* fractions in the ABTS assay were 31.54 µg/mL (F1), 36.26 µg/mL (F2), and 44.36 µg/mL (F3). For *C. vulgaris*, at a concentration of 80 µg/mL, the highest inhibition activity was observed in fractions 1, 2, and 3, while, at a concentration of 40 µg/mL, only fractions 1 and 2 showed a similar inhibition activity (*p* < 0.05). The respective activities were 82% ± 2.04 (fraction 1, 80 µg/mL), 76 ± 2.98% (fraction 2, 80 µg/mL), 75 ± 1.90% (fraction 3, 80 µg/mL), 79 ± 0.98% (fraction 1, 40 µg/mL), and 79 ± 2.05% (fraction 2, 40 µg/mL). The IC_50_ values for *C. vulgaris* fractions in the ABTS assay were 23.44 µg/mL (F1), 21.42 µg/mL (F2), and 30.63 µg/mL (F3). Ascorbic acid showed an antioxidant activity of 91 ± 1.23% at a concentration of 80 µg/mL, with an IC_50_ value of 24.92 µg/mL. In the comparison between the two microalgae species, fraction 1 showed the highest activity in both species, while the lowest activity was observed in fraction 3 of *S. platensis* (*p* < 0.05), as shown in Figure 1B. The activities observed in this study are comparable and, in some cases, exceeded those reported for microalgal protein hydrolysates in previous research [9,32,33]. For instance, an ABTS radical scavenging effect of 72.54 ± 18.16% was reported for a *Chlorella* mix 3 kDa permeate using an in vitro bioassay [8].

The potential iron-chelating activity, determined via FRAP assay, of the peptide fractions extracted from *S. platensis* and *C. vulgaris* are presented in Tables S1 and S2. The values indicate that both the concentration and size of fractions significantly influenced the antioxidant activity (*p* < 0.05). The highest antioxidant activity for *S. platensis* and *C. vulgaris* was obtained for fractions 1 and 2 at a concentration of 80 µg/mL. The values were 74 ± 0.12%, 70 ± 0.12%, 66 ± 0.21%, and 61 ± 0.18%, respectively (*p* < 0.05). The IC_50_ values for *S. platensis* fractions in the FRAP assay were 31.28 µg/mL (F1), 34.76 µg/mL (F2), and 49.93 µg/mL (F3). For *C. vulgaris*, the IC_50_ values were 41.56 µg/mL (F1), 50.09 µg/mL (F2), and 28.87 µg/mL (F3). Vitamin C as a positive control showed an antioxidant activity of 87 ± 0.24% at a concentration of 80 µg/mL, with an IC_50_ value of 7.97 µg/mL. Statistical analysis between the two microalgae species showed that fractions 1 and 2 of *S. platensis* exhibited the highest inhibitory activity (*p* < 0.05), as shown in Figure 1C. These findings are consistent with previous reports of iron-chelating activity in microalgae [32,34]. The FRAP assay results demonstrate significant iron-reducing power in the <3 kDa fractions, suggesting potential applications as food preservatives and in enhancing iron bioavailability [35,36].

The observed variations in antioxidant activity across studies highlight the influence of factors like protein source, enzyme type, and hydrolysis conditions on peptide properties [37,38]. The smaller peptide size achieved through alcalase hydrolysis in this study likely contributed to the enhanced antioxidant activity observed, as smaller peptides often exhibit greater free radical scavenging capacity [38].

### 2.4. Anti-Aging Skin Activities

**Figure 2:**
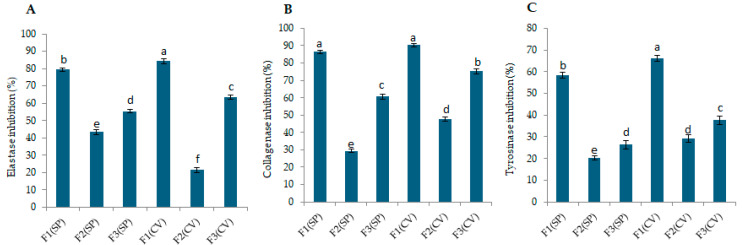
Inhibition of skin-aging enzymes by *S. platensis* (SP) and *C. vulgaris* (CV) peptide fractions (80 µg/mL). Fractions are defined as follows: F1 (<3 kDa), F2 (3–10 kDa), and F3 (>10 kDa). (**A**) Elastase inhibition, (**B**) collagenase inhibition, and (**C**) tyrosinase inhibition. Data are mean ± SD (*n* = 3). Different letters above the bars indicate statistically significant differences between mean values of fractions within each assay (*p* < 0.05).

The anti-aging potential of the *S. platensis* and *C. vulgaris* peptide fractions was assessed by evaluating their ability to inhibit elastase, collagenase, and tyrosinase activity. The elastase inhibition levels of peptide fractions extracted from *S. platensis* and *C. vulgaris* are shown in Table S3. Concerning fractions of *S. platensis*, the fraction < 3 kDa showed significantly higher elastase inhibition activity (79.25 ± 0.88%) compared to the other two fractions (*p* < 0.05). Similar findings were observed for *C. vulgaris*; the fraction < 3 kDa exhibited the highest elastase inhibition activity (84.43 ± 1.45%) compared to the other two fractions. Statistical analysis comparing the two microalgae species also showed that fraction 1 of *C. vulgaris* had significantly higher activity compared to the other groups (*p* < 0.05), as shown in Figure 2A.

The collagenase inhibition activity of the fractions is shown in Table S3. Higher activity (86.21 ± 0.97%) was observed for *S. platensis* in the fraction smaller than 3 kDa. Peptides within this fraction significantly (*p* < 0.05) exhibited higher activity compared to the other two fractions. Similarly, in *C. vulgaris*, the fraction smaller than 3 kDa showed significantly (*p* < 0.05) higher collagenase inhibition activity (90.52 ± 0.87%) compared to the others. Statistical analysis between the two microalgae species showed that fraction 1 in both species had the highest activity (*p* < 0.05), as shown in Figure 2B.

The potential of peptide fractions for tyrosinase inhibition is presented in Table S3. In *S. platensis*, peptide fractions smaller than 3 kDa exhibited the highest tyrosinase inhibition activity (58.22 ± 1.43%). Concerning *C. vulgaris*, the fraction smaller than 3 kDa showed the highest tyrosinase inhibition activity (66.12 ± 1.52%), with a statistically significant difference (*p* < 0.05) compared to the other two fractions. Statistical comparison between the two species also showed that fraction 1 of *C. vulgaris* had the highest tyrosinase inhibitory activity (*p* < 0.05).

These results highlight the significant anti-aging potential of peptide fractions derived from *S. platensis* and *C. vulgaris*, particularly the < 3 kDa peptide fractions (80 µg/mL), which demonstrated superior inhibition of elastase (79.25 ± 0.88% for *S. platensis* and 84.43 ± 1.45% for *C. vulgaris*), collagenase (86.21 ± 0.97% for *S. platensis* and 90.52 ± 0.87% for *C. vulgaris*), and tyrosinase (58.22 ± 1.43% for *S. platensis* and 66.12 ± 1.52% for *C. vulgaris*) compared to fractions with larger peptides. The findings can be compared with previous research on natural compounds, such as Kae-3-Rob from the aquatic medicinal plant *Nelumbo nucifera*, which exhibited tyrosinase inhibition (69.84 ± 6.07%), collagenase inhibition (58.24 ± 8.27%), and elastase inhibition (26.29 ± 7.16%) [39]. While synthetic inhibitors like kojic acid (10 µM, 1.42 µg/mL), a suggested positive control for tyrosinase, showed 51.2 ± 0.9% inhibition, oleanolic acid (10 µM, 4.57 µg/mL), for elastase, showed 46.7 ± 1.5% inhibition, and 1,10-Phenantroline (100 µM, 18.02 µg/mL) inhibited collagenase by 33.4 ± 1.9% [39]. While direct comparisons with synthetic inhibitors are challenging due to differing assay conditions and concentrations, the observed enzyme inhibition levels are encouraging, particularly considering the natural origin of the peptides. These findings corroborate previous studies reporting similar enzyme inhibitory activities in microalgal extracts and protein hydrolysates [25,32,40].

### 2.5. Identification of Peptide Using High-Resolution Tandem Mass Spectrometry and Molecular Networking

**Figure 3:**
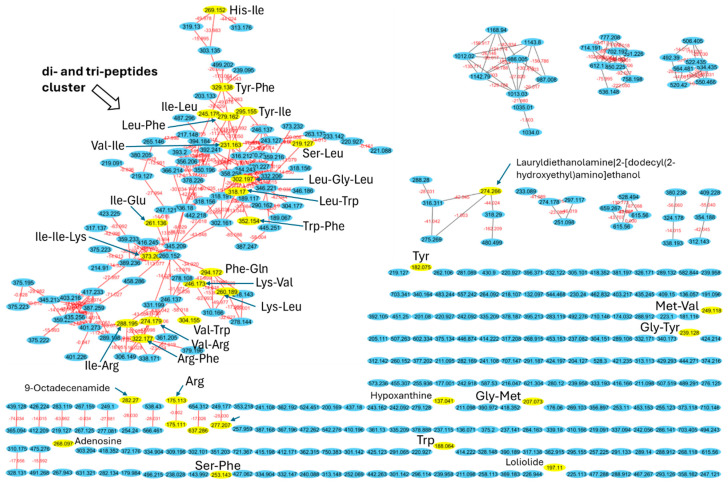
Molecular network of F1 peptide fraction (<3 kDa) of *S. platensis* and *C. vulgaris* hydrolyzed proteins. Each node represents a consensus MS/MS spectrum, and edges between the nodes represent spectral similarity as determined using the cosine score. Nodes with assigned labels were annotated from GNPS MS/MS library searches.

To investigate the composition of the F1 peptide fraction (<3 kDa) of *S. platensis* and *C. vulgaris* hydrolyzed proteins, high-performance liquid chromatography coupled with high-resolution tandem mass spectrometry (HPLC-MS/MS) was performed. The resulting data were used to construct a molecular network using the GNPS platform [41] of all metabolites in each sample, with spectra from blank measurements filtered out before MS/MS networking. The resulting network illustrates the structural similarities among metabolites and, by comparing the fragmentation patterns of each metabolite to a library of known metabolites, enables the annotation of the metabolites present in the samples. Molecular networking and annotation of metabolites revealed that the majority of the metabolites are of peptidic origin. The network comprises 365 nodes, with the largest cluster containing 99 nodes (Figure 3). Many of the library hits within this cluster are di- and tri-peptides, such as Ile-Arg, Lys-Leu, Leu-Phe, Ile-Leu, His-Ile, Tyr-Phe, Ile-Ile-Lys, and Leu-Gly-Leu (Figure 3 and Table S4). This cluster exhibits a significant diversity of di- and tri-peptides, with 99 nodes differing in mass and interconnected by 190 edges (red lines). The masses (*m*/*z*) range from 189.067 to 499.202 Da, with various mass shifts corresponding to different amino acids or chemical moieties, confirming their di- and tri-peptide structures. Additionally, the analysis detected several single amino acids, such as tyrosine, tryptophan, and arginine, as well as adenosine and hypoxanthine.

**Figure 4:**
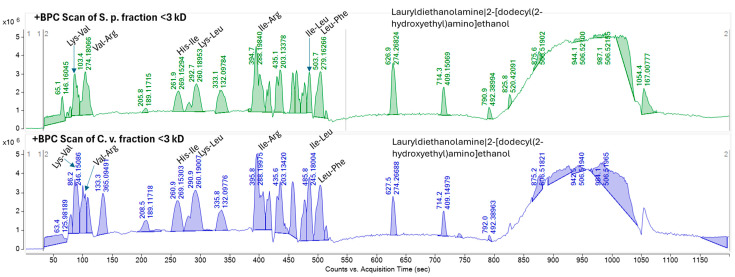
Base peak chromatograms (BPCs) of F1 peptide fraction (<3 kDa) of *S. platensis* and *C. vulgaris* hydrolyzed proteins. BPC-annotated peaks are based on GNPS molecular networking.

**Figure 5:**
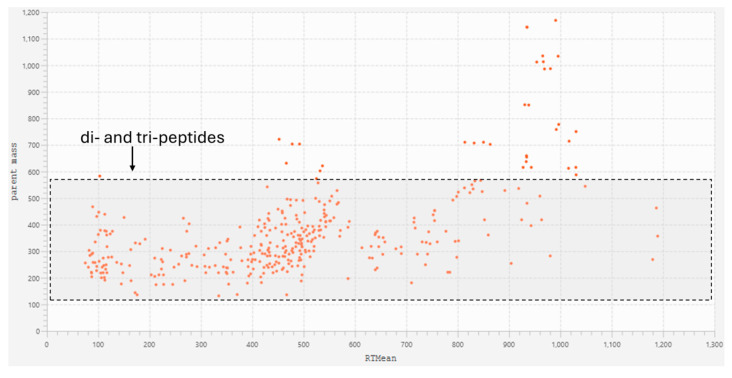
Scatter plot of metabolite mass versus their retention time in the molecular network of the F1 peptide fraction (<3 kDa) of *S. platensis* and *C. vulgaris* hydrolysates (corresponding to Figure 3). Dipeptides and tripeptides within the mass range from 115 to 559 Da are highlighted in a gray box with a black dashed outline. This category includes 328 out of 365 nodes.

Comparing the molecular network with the base peak chromatograms (BPCs) of each extract displays the signal intensity of the most intense mass peak in the MS spectra at any given retention time point (*x*-axis) during the LC-MS run. The BPCs of both extracts from *S. platensis* and *C. vulgaris* were similar, with one additional peak in *C. vulgaris* (RT: 133.3 s). It is evident that the majority of the metabolites within the extracts with molecular weights smaller than 3 kDa are di- and tri-peptides (Figure 4). Most of these metabolites appeared at retention times ranging from 87.117 to 570.315 s, demonstrating both hydrophilic and lipophilic properties. Several annotated di- and tri-peptides were detected as major compounds in the BPCs of both extracts from *S. platensis* and *C. vulgaris*, including Lys-Val, Val-Arg, His-Ile, Lys-Leu, Ile-Leu, and Leu-Phe (Figure 4). A scatter plot of metabolite mass versus retention time showed that the majority of detected metabolites (328 out of 365 nodes), identified as di- and tripeptides, fell within a mass range from 115.0502 *m*/*z* (Gly-Gly) to 559.2458 *m*/*z* (Trp-Trp-Trp) (Figure 5).

While direct clinical evidence for the skincare benefits of every single identified peptide is limited, the presence of these small peptides and their constituent amino acids is significant. Many of these amino acids are known to play crucial roles in skin health, including collagen production (e.g., Arg, Gly, Tyr), antioxidant defense (e.g., Tyr, Trp, Phe), and skin barrier function (e.g., Trp) [42,43]. Notably, the prominent presence of the Lys-Leu dipeptide in the < 3 kDa fractions of both microalgae suggests a potential contribution to the observed antioxidant and anti-aging effects. A study by Yokoyama (2023) demonstrated that food-derived bioactive peptides, specifically Lys-Leu and its isomer Leu-Lys, exhibit antioxidant and antiglycation activities, prolonging the lifespan of *C. elegans* and suppressing ROS and superoxide radicals [44]. They suggest that such dipeptides can be used as a novel functional food ingredient. Additionally, the oligopeptides composed of Leu, Val, and Phe showed a high affinity for hydrogen bonding with free radicals, thereby enhancing their radical scavenging efficiency and overall antioxidant capacity [45]. In fact, the <3 kDa fractions were found to contain several dipeptides, including Leu-Phe, Ile-Leu, His-Ile, Val-Arg, and Lys-Val, all of which contribute to these observed effects. While Aurino (2025) [10] found larger peptides with anti-inflammatory and anti-diabetic potentials, our focus was on smaller peptides for skin applications. Both studies, notably, identified peptides containing hydrophobic amino acids (e.g., Leu, Phe, Ile) that were frequently present, known to be important for bioactivity [10]. The small size of these di- and tri-peptides may also facilitate better skin penetration. The identification of these peptides provides a strong foundation for future research aimed at elucidating their specific mechanisms of action and validating their efficacy in skincare applications.

In summary, this study offers a novel approach to harnessing the bioactive potential of *S. platensis* and *C. vulgaris*, demonstrating the successful isolation and characterization of peptide fractions with significant antioxidant and anti-aging activities. The identification of specific di- and tri-peptides through advanced LC-MS/MS and molecular networking techniques not only provides a molecular basis for these bioactivities but also establishes a foundation for targeted peptide design. This work pioneers the application of comprehensive peptidomics to marine microalgae, revealing a rich reservoir of natural compounds with the potential for new innovation in cosmeceuticals and nutraceuticals.

## 3. Materials and Methods

### 3.1. Algae Cultivation and Species Selection

The microalgae species *S. platensis* and *C. vulgaris*, known for their high protein content, were selected as suitable candidates for bioactive peptide production. These species were obtained from the Gil-Fouga microalgae company (Rasht, Iran). They were cultivated separately in their specific culture media [46,47]. Cultivation was conducted in 5 L Erlenmeyer flasks containing 1 L of media. The growth of the microalgae was monitored daily using a Neubauer chamber under a microscope. In addition, the absorbance at 750 nm was measured using a spectrophotometer. Upon the logarithmic growth phase, the cells were harvested through centrifugation at 10,000× *g* for 15 min at 4 °C using a refrigerated centrifuge (Universal, 320R, Tuttlingen, Germany). The biomass was washed three times with sterile distilled water and then lyophilized using a freeze-dryer (Operan, FDU-7012, Gyeonggi, South Korea). The obtained lyophilized powder was stored at −20 °C for subsequent analyses.

### 3.2. Chemical Analysis

Standard methods were used to determine the chemical composition of *S. platensis* and *C. vulgaris*, including protein, fat, carbohydrate, moisture, and ash content. The moisture content was measured using an oven at 105 °C for 3 h. The ash content was determined by incinerating the samples in a furnace at 625 °C for 6 h. The fat content was measured using the Soxhlet extraction method using hexane–chloroform solvent. Protein content was determined using the automatic Kjeldahl method, which involves digestion with H_2_SO_4_/CuSO_4_, followed by distillation and titration of the evolved NH_3_ [48]. The carbohydrate content for each sample was calculated by difference using the following formula:

Carbohydrate = 100 − (Fat + Protein + Ash + Moisture)

### 3.3. Bioactive Peptides Production

Fat removal: The microalgae powder was homogenized within hexane in a ratio of 1:5 (weight/volume) for fat extraction. This suspension was stirred using a magnetic stirrer for 1 h at laboratory temperature. Subsequently, it was centrifuged at 8000 g for 15 min at 4 °C to separate the powder from the hexane [49].

Cell wall disruption: Algae cell walls were disrupted using a sonicator (BRANSON Sonifier 250, 200 W, 20 kHz, Danbury, CT, USA). Fat-extracted microalgae powder was mixed with distilled water (1:15 ratio) and sonicated in an ice bath to maintain temperature. Sonication was performed for 5 cycles (1 min on, 30 s off) with an output control of 3 and a 20% duty cycle.

Protein isolation: Protein isolation was conducted using an alkaline method. The fat-extracted microalgae powder was mixed with distilled water in a ratio of 1:15 and homogenized using a magnetic stirrer at 1400 rpm for 45 min. The pH of the solution was adjusted to 10 for *S. platensis* and 11 for *C. vulgaris* using NaOH. The mixture was then centrifuged at 2800× *g* for 30 min at 4 °C, and the supernatant was collected for further analyses [48].

Isoelectric precipitation: To precipitate the proteins, the pH of the supernatant obtained from centrifugation was adjusted to 4.5 using HCl (isoelectric point). It was then centrifuged at 10,000× *g* for 20 min. The clear supernatant was discarded, and the recovered protein was further washed twice by centrifugation at 5000× *g* for 5 min each time. Finally, the recovered protein was freeze-dried using a freeze dryer and stored at −20 °C until further use [50].

Enzymatic hydrolysis: Enzymatic hydrolysis of the isolated protein was performed using alcalase 2.4 L (Merck, Saint Louis, MO, USA), extracted from *Bacillus licheniformis*, with an enzymatic activity of 4.2 AU/kg and a density of 1.18 g/mL [51]. The protein isolate was mixed with distilled water in a ratio of 1:15 (weight/volume) and homogenized. Then, the pH and temperature of the solution were adjusted to 8.5 and 55 °C, respectively, suitable for the optimal activity of alcalase. The hydrolysis process was initiated by adding 5% (*w*/*v*) alcalase (weight/volume) and continued for 5 h. To terminate the hydrolysis process, the solution was heated at 90 °C for 5 min. After cooling, the samples were centrifuged at 10,000 g for 20 min, and the supernatant containing bioactive peptides was directly used for peptide purification [52].

### 3.4. Hydrolysis and Peptide Chain Length Measurements

The degree of hydrolysis (DH) was determined using 10% (*v*/*v*) trichloroacetic acid (TCA) precipitation. This method quantifies the ratio of TCA-soluble proteins to total protein. Briefly, an equal volume of protein solution was mixed with the TCA solution and centrifuged at 5000× *g* and 20 °C for 5 min after 10 min of incubation. The protein content in the supernatant (soluble phase) was then measured using the Bradford assay, with bovine serum albumin (BSA) as the standard [52]. The DH was calculated using the equation below:

DH = (Soluble N in sample/Total N in sample) × 100

The approximate peptide chain length (PLC) was calculated from the DH using a previously described method [53] (the following equation):

PLC = 100/DH

where DH is the degree of protein hydrolysis expressed as a percentage.

### 3.5. Peptide Purification

To purify bioactive peptides, hydrolyzed proteins from *S. platensis* and *C. vulgaris* were sequentially passed through Amicon ultra-centrifugal filters (Merck, Darmstadt, Germany). First, the hydrolysates were centrifuged through a 10 kDa filter. The flow-through was then passed through a 3 kDa filter (centrifuged at 4 °C, 10,000× *g* for 20 min). Peptides retained by the 10 kDa filter were designated as fraction F3 (>10 kDa). The fraction that passed through the 10 kDa filter but was retained by the 3 kDa filter (3–10 kDa) was designated as fraction F2. The fraction that passed through the 3 kDa filter (<3 kDa) was designated as fraction F1. All fractions were freeze-dried and stored at −20 °C until use.

### 3.6. Antioxidant Features

#### 3.6.1. DPPH Assay

To assess the radical scavenging activity of the peptides, a 2,2-diphenyl-1-picrylhydrazyl (DPPH) assay was used based on the method described in [54]. DPPH (4 mg) was dissolved in 100 mL of 95% ethanol. Various concentrations (5, 10, 20, 40, and 80 µg/mL) of the hydrolyzed protein were prepared from fractions F1, F2, and F3. Then, equal volumes of the sample were mixed with the DPPH solution. The resulting solution was shaken for 1 min and then kept at room temperature in the dark for 30 min. Finally, the absorbance of all samples was read at a wavelength of 517 nm using a microplate reader. The scavenging activity was calculated using the following equation. The DPPH solution with water was used as the blank, and ascorbic acid at a concentration of 80 µg/mL was used as the positive control.

Free radical scavenging (%) = (A0 − A1)/A0 × 100

where A0 was the blank absorbance and A1 was the sample absorbance.

#### 3.6.2. ABTS Assay

The 2,2′-azinobis-3-ethylbenzothiazoline-6-sulfonic acid (ABTS) test was performed using a slightly modified method discussed in [55]. Different concentrations (5, 10, 20, 40, and 80 µg/mL) of the hydrolyzed protein from fractions F1, F2, and F3 were prepared, and then 7 mM ABTS solution was mixed with 2.54 mM potassium persulfate. The mixture was kept in the dark at room temperature for 16 h. The ABTS radical solution was then diluted with potassium phosphate buffer (pH 7.4) to reach an absorbance of 0.70 ± 0.02 at 734 nm for the assay. Then, the ABTS solution (980 µL) was mixed with 20 µL of each sample concentration, and after 10 min, the absorbance was measured at 734 nm using a microplate reader. The ABTS radical scavenging activity was determined using the following formula. The ABTS solution with water was used as the control, and ascorbic acid at a concentration of 80 µg/mL was used as the standard.

Free radical scavenging (%) = (A0 − A1)/A0 × 100

where A0 was the control absorbance and A1 was the sample absorbance.

#### 3.6.3. FRAP Assay

To conduct the ferric reducing antioxidant power (FRAP) assay, various concentrations of hydrolyzed protein (5, 10, 20, 40, and 80 µg/mL) from fractions F1, F2, and F3 were prepared. Then, 1 mL of the sample was mixed with 2.5 mL of 0.2 M potassium phosphate buffer (pH 6.6) and 2.5 mL of 1% potassium ferricyanide. The mixture was incubated at 50 °C for 20 min, and then 2.5 mL of 10% trichloroacetic acid (TCA) was added to the mixture. The mixture was centrifuged at 10,000× *g* for 10 min. Then, 500 µL of the supernatant was mixed with 500 µL of distilled water and 100 µL of 0.1% ferric chloride (FeCl_3_). The solution was then incubated at a constant temperature for 30 min until it turned green. The absorbance of the solution was measured at 700 nm using a microplate reader [56]. Ascorbic acid at a concentration of 80 µg/mL was used as the standard.

Where activities greater than 50% were observed, IC_50_ values were also determined by plotting the activity percentage values as a function of the sample concentration and solving the obtained function for 50% activity [10].

### 3.7. Evaluation of Skin Anti-Aging Potential

#### 3.7.1. Elastase Inhibition Assay

The anti-elastase activity of the hydrolyzed peptides was evaluated according to the method of Honda [57]. To this end, 40 µL of the sample was mixed with 25 µL of a 50 mU/mL solution of human neutrophil elastase and 50 µL of Suo(OME)-Ala-Ala-Pro-Val-MCA solution. The solution was stirred at 270 rpm for 10 s. The fluorescence intensity was then measured using a microplate reader at Ex/Em 360/465 nm. After incubation at 37 °C for 60 min, the fluorescence intensity (Ex/Em 360/465 nm) was measured again. The anti-elastase activity of the samples was determined based on the fluorescence intensity of the solutions before and after incubation. Oleanolic acid (10 µM or 4.57 µg/mL) is suggested as a positive control for elastase inhibition [39].

#### 3.7.2. Collagenase Inhibition Assay

The anti-collagenase activity of the obtained peptides was evaluated according to the proteolytic degradation between collagenase and the synthetic substrate FALGPA (N-(3-[2-Furyl]-acryloyl)-Leu-Gly-Pro-Ala). This activity was assessed at a wavelength of 345 nm in the presence of collagenase inhibitors [58]. To perform the assay, 0.25 units per mL of collagenase from *Clostridium histolyticum* was reacted with 40 µL of the sample and 20 µL of 50 mM Tris buffer (pH 7.5, containing 100 mM calcium chloride and 5 mM sodium chloride) for 15 min. After pre-incubation, 40 µL of a 2 mM FALGPA solution was added to each well, and the absorbance was measured after 20 min incubation. 1,10-Phenantroline (100 µM, 18.02 µg/mL) is suggested as a positive control for collagenase inhibition [39].

#### 3.7.3. Tyrosinase Inhibition Assay

The anti-tyrosinase activity of the peptide fractions was evaluated using L-3,4-dihydroxyphenylalanine (L-DOPA) as a substrate [59]. Initially, 40 µL peptide, 40 units per mL of tyrosinase solution, and 100 µL of 100 mM phosphate buffer (pH 6.8) were mixed in 96-well plates and incubated at 23 °C for 3 min. Then, 50 µL of a 2.5 mM L-DOPA solution was added, and the resulting solution was shaken at 270 rpm for 10 s. The optical density at 490 nm was measured using a microplate reader. After incubating the solutions at 23 °C for 10 min, the absorbance at 490 nm was measured again. The anti-tyrosinase activity of the samples was calculated based on the absorbance values at 490 nm before and after incubation. Kojic acid (10 µM, 1.42 µg/mL) is suggested as a positive control for tyrosinase inhibition [39].

### 3.8. High-Pressure Liquid Chromatography Tandem Mass Spectrometry (HPLC-MS/MS)

For mass spectrometry analysis, the lyophilized samples were firstly mixed with 2 mL MeOH (LCMS grade) to dissolve the non-polar components. The resulting heterogeneous solution (yellowish) was allowed to decant, and the supernatant was taken into a new 2 mL glass vial and dried through a rotatory evaporator (39 °C). Then, the residue was dissolved with MeOH to reach a 0.5 mg/mL final concentration. The solution was filtered through a 0.22 um PTFE filter. This solution was then transferred into an LC vial for subsequent analysis through LCHRMSMS. Mass spectrometry data were recorded on an Agilent 6550 iFunnel^®^ Q-TOF LC/MS system (Santa Clara, CA, USA) used with an Agilent Jet Stream^®^ source. The chromatographic separation was performed on an Agilent 1200 HPLC system, which had a Waters Atlantis T3 column (5 µm, 4.6 × 50 mm). The separation was performed in a temperature-controlled oven at 20 °C using a gradient of water (A) and acetonitrile (B), both supplemented with 0.1% of formic acid. The chromatographic method for all the samples was as follows; t = 0 min, A = 100%, B = 0%; t = 2 min, A = 100%, B = 0%; t = 12 min, A = 0%, B = 100%; t = 14 min, A = 0%, B = 100%; t = 16 min, A = 100%, B = 0%; t = 20 min, A = 100%, B = 0%. The flow rate was 0.5 mL/min. The data were acquired over a range from 50 to 3200 *m*/*z* in positive mode. The fragmentation of the analytes was performed using the auto MS/MS function of the instrument, where the parameters for fragmentation were related through a function between *m*/*z* and collision energy (CE, KeV). The function was performed with the following points: *m*/*z* 100, CE = 16; *m*/*z* 300, CE = 24, *m*/*z* 500, CE = 30; *m*/*z* 1000, CE = 40; *m*/*z* 1500, CE = 50; *m*/*z* 2000, CE = 70. The fragmentation was carried out with a frequency of 3 Hz for all ions over a threshold of a relative abundance of 500 in the TIC level. The injection volume for all of the samples was 5 uL.

### 3.9. Molecular Networking

The MS/MS data were converted to mzXML format using MSConvert (Version: 3.0) and transferred to the Global Natural Product Social Molecular Networking (GNPS) server (https://gnps.ucsd.edu/, accessed on 1 February 2025) [36]. A molecular network was created using the online workflow at GNPS using the spectra with a minimum of four fragment ions and by merging all identical spectra into nodes, representing parent masses. Compounds with similar fragmentation patterns are connected by edges, displaying molecular families with similar structural features. The data were filtered by removing all MS/MS peaks within + /− 17 Da of the precursor *m*/*z*. MS/MS spectra were window-filtered by choosing only the top 6 peaks in the + /− 50 Da window throughout the spectrum. The resulting data were then clustered using MS-Cluster with a parent mass tolerance of 0.02 Da and an MS/MS fragment ion tolerance of 0.02 Da to create consensus spectra. Further, consensus spectra that contained less than 2 spectra were discarded. A network was then created where edges were filtered to have a cosine score above 0.6 and more than 6 matched peaks. Further edges between two nodes were kept in the network if and only if each of the nodes appeared in each other’s respective top 10 most similar nodes. The spectra in the network were then searched against GNPS spectral libraries. The library spectra were filtered in the same manner as the input data, including the analog search. All matches kept between network spectra and library spectra were required to have a score above 0.5 and at least four matched peaks. The network was visualized using Cytoscape 3.9.1 (Seattle, WA, USA).

### 3.10. Statistical Analysis

This study was conducted using a completely randomized design. Statistical analysis of the data was performed using SPSS 20 software. The normality of the data was assessed using the Kolmogorov–Smirnov test. Depending on the experimental design, one-way analysis of variance (ANOVA) was conducted, followed by Duncan’s multiple range test for post-hoc comparisons. Independent samples t-tests were used for two-group comparisons. Statistical significance was set at *p* < 0.05.

## 4. Conclusions

This study demonstrated that *Spirulina platensis* and *Chlorella vulgaris* are rich sources of bioactive peptides with significant antioxidant and anti-aging potential. Enzymatic hydrolysis with alcalase yielded predominantly <3 kDa peptides, which exhibited strong DPPH, ABTS, and FRAP activities, as well as the effective inhibition of elastase, collagenase, and tyrosinase. The identification of specific di- and tri-peptides, such as Lys-Val, Val-Arg, His-Ile, and Leu-Gly-Leu, through advanced LC-MS/MS and molecular networking techniques provides a molecular basis for these bioactivities and establishes a foundation for targeted peptide design. However, to fully validate the benefits of these microalgal peptides for skin health and anti-aging, further in vitro and in vivo studies, along with proper positive controls, are essential. Additionally, scalable production methods and innovative delivery systems should be explored to maximize their therapeutic impact and commercial viability.

In conclusion, the high yields of these small peptides, coupled with their observed bioactivities, underscore the potential of *S. platensis* and *C. vulgaris* as valuable resources for the food, pharmaceutical, and cosmetic industries. These findings highlight the promise of these microalgae as sustainable and natural sources of marine bioactive compounds for anti-aging and antioxidant applications, demonstrating (bio)technological innovation in the development of added-value products from marine microalgae.

## Figures and Tables

**Table 1:** Chemical analysis of microalgae dry materials (A), after fat removal (B), isolated protein (C), degree of hydrolysis and peptide chain length of enzymatic hydrolysis (D), and peptide content of purified fractions (E).

			*S. platensis*	*C. vulgaris*
A	Dry weight (g/L)		1 ± 0.08 ^a^	0.8 ± 0.12 ^a^
	Dry weight (%)		0.1 ± 0.08 ^a^	0.08 ± 0.12 ^a^
	Protein (%)		64 ± 1.45 ^a^	54 ± 0.88 ^b^
	Lipid (%)		10 ± 0.88 ^a^	9 ± 0.90 ^a^
	Moisture (%)		9 ± 0.72 ^a^	5 ± 0.60 ^a^
	Ash (%)		11 ± 0.50 ^a^	14 ±0.78 ^a^
B	Lipid after defat (%)		2.36 ± 0.23 ^a^	3.26 ± 0.35 ^a^
C	Isolated protein (%)		82 ± 0.92 ^a^	72 ± 0.65 ^b^
D	Degree of hydrolysis (%)		33 ± 0.70 ^a^	30 ± 1.19 ^a^
	Length of the peptide chain		3.07 ± 0.08 ^b^	3.33 ± 0.02 ^a^
E	Peptide fraction (mg/g of hydrolyzed protein)	>10 kDa	100 ± 7.50 ^c^	100 ± 3.78 ^c^
		3–10 kDa	200 ± 6.08 ^b^	150 ± 4.93 ^b^
		<3 kDa	650 ± 9.82 ^a^	700 ± 6.17 ^a^

Data are presented as the mean ± standard deviation of triplicate. Different superscript letters within each row indicate statistically significant differences between mean values (*p* < 0.05). Peptide chain length is shown as average amino acid residues.

## Data Availability

The generated data are available from the corresponding author and all data are presented in this paper.

## Supplementary Materials

The following supporting information can be downloaded at: https://www.mdpi.com/article/10.3390/md23040162/s1, Figure S1. A: *S. platensis* cell number, B: Growth curves of *S. platensis* via spectrophotometer, C: *C. vulgaris* cell number, D: growth curves of *C. vulgaris* via spectrophotometer.; Table S1. Antioxidant activity of *S. platensis* (%) based on DPPH, ABTS, and FRAP assays.; Table S2. Antioxidant activity of *C. vulgaris* (%) based on DPPH, ABTS, and FRAP assays.; Table S3: Skin aging-related enzyme inhibitory effects of *S. platensis* and *C. vulgaris*.; Table S4. List of annotated di- and tri-peptides identified using GNPS molecular networking in extracts of *S. platensis* and *C. vulgaris* with molecular weights smaller than 3 kDa and a comparison of the hits against the GNPS library.
