# Supplementary material for: Identification and Evaluation of Antioxidant and Anti-Aging Peptide Fractions from Enzymatically Hydrolyzed Proteins of Spirulina platensis and Chlorella vulgaris
Source: Mar Drugs. 2025 Apr 8;23(4):162. doi: 10.3390/md23040162 (PMC12028799; doi:10.3390/md23040162)
Supplement: Supplementary file 1 [file marinedrugs-23-00162-s001.zip › marinedrugs-3529625-supplementary.pdf]

marine drugs

Article

Supplementary Information

Identification and Evaluation of Antioxidant and Anti-Aging Peptide Fractions from Enzymatically Hydrolyzed Proteins of *Spirulina platensis* and *Chlorella vulgaris*

Baran Masoumifeshani [1], Abdolmohammad Abedian Kenari [1,*], Ignacio Sottorff [2], Max Crüsemann [2,3] and Jamshid Amiri Moghaddam [4,*]

[1] Aquaculture Department, Natural Resources and Marine Science Faculty, Tarbiat Modares University, 46417-76489 Noor, Mazandaran, Iran
[2] Institute for Pharmaceutical Biology, University of Bonn, 53115 Bonn, Germany
[3] Institute of Pharmaceutical Biology, Goethe University Frankfurt, 60438 Frankfurt am Main, Germany
[4] Institute of Biotechnology, RWTH Aachen University, 52074 Aachen, Germany
* Correspondence: aabedian@modares.ac.ir (A.A.K.); j.amiri@biotec.rwth-aachen.de (J.A.M.); Tel.: +98-1144998000-3 (A.A.K.); +49-241-80-21266 (J.A.M.)

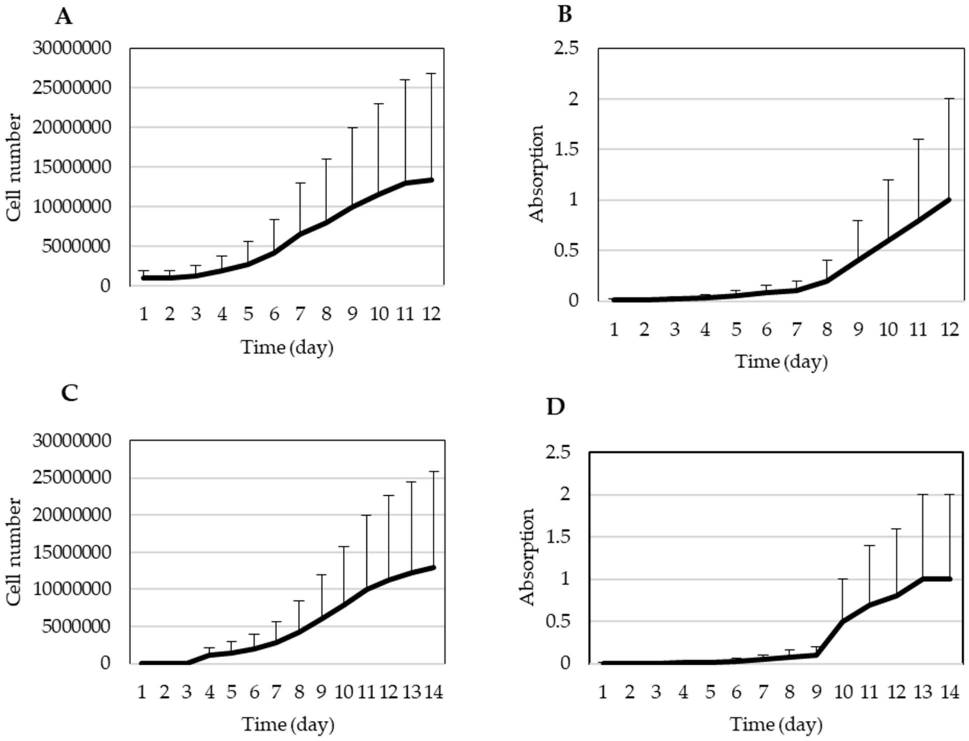

Figure S1. A: *S. platensis* cell number, B: Growth curves of *S. platensis* via spectrophotometer, C: *C. vulgaris* cell number, D: Growth curves of *C. vulgaris* via spectrophotometer. All data are presented as mean ± standard deviation of triplicate.

Table S1. Antioxidant activity of *S. platensis* (%) based on DPPH, ABTS, and FRAP assays.

Concentration (µg/ml)	Fractions	DPPH	ABTS	Iron reduction power
**5**	F1	18±2.50^{f}	23±1.58^{fgh}	31±0.15^{fg}
	F2	23±3.58^{ef}	22±3.76^{fgh}	30±0.15^{fgh}
	F3	22±1.69^{ef}	17±3.24^{h}	25±0.12^{h}
**10**	F1	25±1.86^{ef}	31±2.11^{ef}	40±0.19^{e}
	F2	27±2.63^{ef}	21±1.17^{gh}	33±0.18^{f}
	F3	18±3.53^{f}	27±2.25^{fg}	26±0.07^{gh}
**20**	F1	46±1.45^{d}	45±3.91^{d}	45±0.06^{d}
	F2	45±1.27^{d}	44±3.61^{d}	49±0.12^{cd}
	F3	29±2.24^{ef}	39±2.28^{de}	35±0.0.9^{ef}
**40**	F1	73±2.89^{ab}	69±2.96^{b}	59±0.11^{b}
	F2	67±0.52^{bc}	69±2.23^{b}	58±0.14^{b}
	F3	46±0.76^{d}	58±3.51^{c}	51±0.12^{cd}
**80**	F1	78±3.15^{a}	80±1.51^{a}	74±0.12^{a}
	F2	77±5.65^{a}	74±3.70^{ab}	70±0.12^{a}
	F3	61±0.42^{c}	66±4.14b^{c}	63±0.09^{b}
**IC50**	F1	33.11±1.16^{a}	31.54±0.65^{a}	31.28±0.70^{a}
	F2	33.98±0.60^{a}	36.26±1.82^{b}	34.76±1.17^{b}
	F3	56.87±2.33^{b}	44.36±1.96^{c}	49.92±0.68^{c}
**Control (+)**		91±2.29	91±1.23	87±0.24
**IC50**		20.13±1.31	24.92±0.54	7.98±0.69

F1: Fraction smaller than 3 kD, F2 Fraction between 3-10 kD, and F3 Fraction > 10 kD. Positive control: Vitamin C at a concentration of 80 µg/ml. All data are presented as mean ± standard deviation of triplicate. Different letters in each column indicate statistically significant differences ($p < 0.05$)

Table S2. Antioxidant activity of *C. vulgaris* (%) based on DPPH, ABTS, and FRAP assays.

**Concentration (μg/ml)**	**Fractions**	**DPPH**	**ABTS**	**Iron reduction power**
**5**	F1	21±1.52^{fg}	22±1.31^{g}	23±0.10^{h}
	F2	20±4.87^{fg}	29±1.35^{f}	22±0.25^{h}
	F3	17±2.3^{g}	18±1.36^{g}	20±0.26^{h}
**10**	F1	29±3.98^{e}	35±2.44^{ef}	35±0.27^{efg}
	F2	30±2.57^{e}	37±1.31^{e}	31±0.20^{g}
	F3	25±2.38^{ef}	34±2.41^{ef}	32±0.26^{fg}
**20**	F1	43±2.78^{d}	60±2.48^{c}	47±0.29^{d}
	F2	43±0.90^{d}	59±2.77^{c}	39±0.10^{e}
	F3	38±1.99^{d}	52±2.78^{d}	38±0.19^{ef}
**40**	F1	64±2.78^{b}	79±0.98^{ab}	53±0.06^{c}
	F2	62±2.30b^{c}	79±2.05^{ab}	51±0.17^{cd}
	F3	55±2.44^{c}	72±3.00^{b}	46±0.20^{d}
**80**	F1	77±1.32^{a}	82±2.04^{a}	66±0.21^{a}
	F2	73±0.37^{a}	76±2.98^{ab}	61±0.18^{ab}
	F3	59±1.19b^{c}	75±1.90^{ab}	57±0.30^{d}
**IC50**	F1	35.36±1.75^{a}	23.44±1.19^{a}	41.56±1.93^{b}
	F2	37.21±1.26^{a}	21.42±1.35^{a}	50.09±1.17^{c}
	F3	52.02±2.94^{b}	30.63±0.31^{b}	58.19±1.17^{c}
**Control (+)**		91±2.29	91±1.23	87±0.24
**IC50**		20.13±1.31	24.92±0.54	7.98±0.69

F1: Fraction smaller than 3 kD, F2 Fraction between 3-10 kD, and F3 Fraction > 10 kD. Positive control: Vitamin C at a concentration of 80 μg/ml. All data are presented as mean ± standard deviation of triplicate. Different letters in each column indicate statistically significant differences ($p < 0.05$)

Table S3: Skin aging-related enzyme inhibitory effects of *S. platensis and C. vulgaris.*

Species	Fractions	Elastase inhibition (%)	Collagenase inhibition (%)	Tyrosinase inhibition (%)
S. platensis	F1	79.25±0.88[a]	86.21±0.97[a]	58.22±1.43[a]
	F2	43.36±1.45[c]	29.64±0.88[c]	20.39±0.93[c]
	F3	55.45±0.93[b]	60.67±1.22[b]	26.33±2.03[b]
C. vulgaris	F1	84.43±1.45[a]	90.52±0.87[a]	66.12±1.52[a]
	F2	21.45±1.36[c]	47.67±1.22[c]	29.47±1.76[c]
	F3	63.64±1.23[b]	75.38±1.61[b]	37.64±2.03[b]

F1: Fraction smaller than 3 kD, F2 Fraction between 3-10 kD, and F3 Fraction > 10 kD. Positive Control: Vitamin C at a concentration of 80 μg/ml. All data are presented as mean ± standard deviation of triplicate. Different letters in each column indicate statistically significant differences ($p < 0.05$)

Table S4. List of Annotated di- and tri-peptides identified using GNPS molecular networking in extracts of *S. platensis* and *C. vulgaris* with molecular weights smaller than 3 kD, and comparison of the hits against the GNPS library.

Compound name	Parent mass	RTMean	Shared peaks	MassDiff	MZErrorPPM	Spectra in *S. platensis*	Spectra in *C. vulgaris*	Sum(precursor intensity)
Arg	175.111	245	7	0	46	11	9	1063650
Gly-Tyr	239.128	423	6	0	106	0	2	219658
Tyr	182.075	418	6	0	33	1	1	20414.9
Arg-Phe	322.177	418	13	0	31	3	5	3034840
Gly-Met	207.073	393	7	0	34	1	1	110070
His-Ile	269.152	223	8	0	45	8	8	6476530
Ile-Arg	288.195	272	11	0	28	19	23	28000000
Ile-Glu	261.136	415	7	0	46	6	2	5890900
Ile-Ile-Lys	373.268	458	8	0	35	2	3	907111
Ile-Leu	245.178	489	7	0	33	4	5	6523160
Leu-Gly-Leu	302.197	493	6	0	43	2	3	263980
Leu-Phe	279.162	513	6	0	29	4	3	6710350
Leu-Trp	318.17	520	7	0	35	2	4	1666900
L-Tryptophan	188.064	495	6	0	43	1	0	60081.8
Lys-Leu	260.189	225	7	0	31	9	11	8957020
Lys-Val	246.173	111	7	0	32	2	2	940538
Met-Val	249.118	444	6	0	36	2	1	167997
Phe-Gln	294.172	403	9	0	95	2	5	1471660
Ser-Leu	219.127	440	9	0	32	2	1	315250
Ser-Phe	253.143	471	7	0	99	0	1	12518.7
Trp-Phe	352.154	539	6	0	34	0	2	38449.2
Tyr-Ile	295.155	479	7	0	31	2	2	1937350
Tyr-Phe	329.138	490	6	0	39	2	5	718040
Val-Arg	274.179	110	11	0	29	1	3	3451130
Val-Ile	231.163	458	7	0	35	4	4	5559840
Val-Trp	304.155	503	7	0	33	1	1	51678.6
